# Comparative Study of Chemical Compositions and Antioxidant Capacities of Oils Obtained from 15 Macadamia (*Macadamia integrifolia*) Cultivars in China

**DOI:** 10.3390/foods10051031

**Published:** 2021-05-10

**Authors:** Xixiang Shuai, Taotao Dai, Mingshun Chen, Ruihong Liang, Liqing Du, Jun Chen, Chengmei Liu

**Affiliations:** 1 State Key Laboratory of Food Science and Technology, Nanchang University, Nanchang 330047, China; shuaixixiang1989@163.com (X.S.); chenshun1221@163.com (M.C.); liangruihong@ncu.edu.cn (R.L.); chen-jun1986@hotmail.com (J.C.); 2 Agro-Products Processing Science and Technology Research Institute, Guangxi Academy of Agricultural Sciences, Nanning 530007, China; ncubamboo@163.com; 3 South Subtropical Crop Research Institute, China Academy of Tropical Agricultural Sciences, Zhanjiang 524091, China; duliqing927618@163.com

**Keywords:** macadamia oil, cultivars, minor components, antioxidant capacity, triacylglycerols

## Abstract

The planting area of macadamia in China accounted for more than one third of the world’s planted area. The lipid compositions, minor components, and antioxidant capacities of fifteen varieties of macadamia oil (MO) in China were comparatively investigated. All varieties of MO were rich in monounsaturated fatty acids, mainly including oleic acid (61.74–66.47%) and palmitoleic acid (13.22–17.63%). The main triacylglycerols of MO were first time reported, including 19.2–26.1% of triolein, 16.4–18.2% of 1-palmitoyl-2,3-dioleoyl-glycerol, and 11.9–13.7% of 1-palmitoleoyl-2-oleoyl-3-stearoyl-glycerol, etc. The polyphenol, α-tocotrienol and squalene content varied among the cultivars, while Fuji (791) contained the highest polyphenols and squalene content. Multiple linear regression analysis indicated the polyphenols and squalene content positively correlated with the antioxidant capacity. This study can provide a crucial directive for the breeding of macadamia and offer an insight into industrial application of MO in China.

## 1. Introduction

The macadamia (*Macadamia integrifolia*) is an evergreen native tree indigenous to the coastal rainforests of Australia [1]. The macadamia fruit is made up of the husk, shell, and kernel [2]. The kernel is a rich source of lipids, proteins and important micronutrients [3,4]. However, its chemical composition may vary greatly influenced by the cultivar, kernel maturity, geographical location and growth conditions. Castilho Maro, et al. [5] studied the chemical composition of 22 macadamia varieties in Brazil, and the results showed lipid contents of macadamia kernel ranging from 33.13% to 64.28%, and protein from 8.56% to 19.24%. Entelman, et al. [6] characterized eight macadamia varieties in Sao Paulo, and reported that lipid content of macadamia kernel in the range of 65.21–68.48%. Kaijser, et al. [7] investigated the chemical composition of four macadamia cultivars in New Zealand, and found that lipid content of macadamia kernel was 69.1–78.4%.

The minor components of macadamia oil (MO) may also influenced by cultivar and location. Mereles, et al. [8] found the difference in the contents of polyphenols (77.9–96.3 mg gallic acid equivalent/kg) and α-tocopherol (0.2–18.4 mg/100 g) of three macadamia cultivars grown in Paraguay. Gong, et al. [9] reported that the composition and content of tocopherol (22.3–49.7 mg/kg oil) and sterol (1952–2571 mg/kg oil) of 3 MO from the U.S. market were different. Wall [10] indicated tocotrienols and squalene was largely effect by varieties, their content in seven macadamia cultivars produced in Hawaii were 31–92 and 72–171 μg/g oil, respectively.

The introduction of macadamia from Australia into China began in the 1970s. By the end of 2018, the cultivated area of macadamia in China has exceeded 3012.06 km^2^, which accounted for more than one third of the world’s planted area [11]. Up to now, China has become the largest and fastest growing macadamia plantation country in the world. The geographical environment of China is quite different from that of the above mentioned regions, however, the chemical compositions and antioxidant capacities of MOs cultivated in China were never reported.

In this study, the compositions of macadamia kernel, and the fatty acids, triacylglycerols, minor components, as well as antioxidant activity of MO of 15 different varieties of macadamia in China were comparatively investigated. In addition, the correlation between the main minor components and the antioxidant capacity were analyzed by multiple linear regression (MLR) analysis. This study are expected to provide a crucial directive for the industrial application of MO in China.

## 2. Materials and Methods

### 2.1. Material

Fifteen macadamia cultivars (1 = Hinde (H2), 2 = Fuji (791), 3 = Purvis (294), 4 = HAES 816, 5 = Keauhou (246), 6 = Beaumont (695), 7 = Pahala (788), 8 = HAES 863, 9 = A4, 10 = A16, 11 = HAES 344, 12 = Keaau (660), 13 = Makai (800), 14 = GUANG 11, 15 = own choice (OC)) were collected from a commercial plantation and harvested during the 2019 in September. The selected plantation is located in Lincang City (longitude: 99°26′39.2″, latitude: 14°11′43.6″), Yunnan Province, which belongs to the mountainous area of low hot valley and is the largest macadamia-cultivated regions in China. Macadamias were collected using the diagonal method. A total of 10 plants with same growth potential and without disease and pest were randomly selected in ripening period, on the canopy of which 500 g of macadamias were collected, respectively, at lower, middle, higher, inner and outer parts. The samples were fully mixed, packaged with valve bags and labeled, then taken back to laboratory. The fresh and mature macadamias were selected and its peel were removed to obtain the kernel with shell, then it was dried using hot air drying. The shell of dried fruits were removed, then the kernels were oxygen insulation packed and stored at −20 °C for further analyses.

Standards of 37 fatty acid methyl esters, eight tocopherols, phytosterols, squalane, and 5α-cholestane were purchased from Sigma-Aldrich Co., Ltd. (Shanghai, China). Further, 2,2-Diphenyl-1-picrylhydrazyl (DPPH), 2,2′-Azino-bis (3-ethylbenzothiazoline-6-sulfonic acid) diammonium salt (ABTS), chromatographic grade methanol, ethanol, *n*-hexane, and isopropanol were purchased from the National Institute for the Control of Pharmaceutical and Biological Products (Beijing, China). All other reagents were of analytical reagent grade.

### 2.2. Proximate Analysis of Dried Kernel

The ash, moisture, crude protein and lipid content of dried kernel were measured by the hot air drying at 550 °C (AOAC Method 923.03), hot air drying at 120 °C (AOAC Method 990.19), Kjeldahl (N × 6.25) (AOAC Method 979.09) and Soxhlet extraction (AOAC Method 963.15), respectively [12].

### 2.3. Extraction of MO

Macadamias were collected and pretreated according to the method reported by Li, et al. [13]. The dried macadamia kernels were crushed into slurry, and sifted through a 40-mesh sieve. Then, the MOs were extracted from slurry. In brief, the slurry (100 g) and *n*-hexane (600 mL) were mixed at 50 °C for 4.0 h under 500 r/min. Then, the mixture was filtered with a Brinell funnel. Finally, the *n*-hexane was removed by rotary evaporator (Hei-vap Precision, Heidolph Co., Schwabach, Germany) at 50 °C to obtain the MO. The composition and content of MO were further analyzed.

### 2.4. Fatty Acid and Triacylglycerol Composition Analysis

Briefly, 250 mg of MO was mixed with 10.0 mL of *n*-hexane. Then, the 0.3 mL of KOH-CH_3_OH solution (2.0 mol/L) was added for the methylation reaction. This reaction was performed at room temperature for 30.0 min. Then, 8.0 mL of NaHSO4 solution (0.1 mol/L) was added to neutralize the excessive KOH. Na_2_SO_4_ was used to absorb any trace water remaining in the *n*-hexane part. The obtained fatty acid methyl esters were analyzed by a gas chromatograph (GC) system (Agilent 7890A, Agilent Technologies, Santa Clara, CA, USA) equipped with a BPX capillary column (0.25 μm, 60.0 m × 0.22 mm) and a flame ionization detector (FID) (Agilent Technologies, USA). The following parameters were used: flow rate of nitrogen = 1.0 mL/min; the initial temperature of the column = 60 °C, temperature programming to 170 °C = 10 °C/min, continued temperature programming to 230 °C = 3 °C/min, then held for 15.0 min, injection volume = 1.0 μL, injector temperature = 225 °C, and detector temperature = 250 °C. Fatty acid composition of oils were identified by comparing with the relative retention time of the fatty acid methyl ester peaks with standards of fatty acid methyl esters, and the results were expressed as a percentages of each fatty acid in the total.

Triacylglycerol composition of MO was analyzed using the method of American Oil Chemists’ Society [14]. Triacylglycerol was identified by comparing the retention time, carbon numbers of triacylglycerol standards, and the results were expressed as relative proportion.

### 2.5. Tocopherols Analysis

Tocopherols were analyzed using a high-performance liquid chromatographic system (Agilent 1260, Agilent Technologies, USA) equipped with a C_18_ column (4.6 mm × 250 mm, 5 μm, Agilent Technologies, USA) and ultraviolet detector. Initially, 0.1 g of MO was mixed with 10.0 mL of *n*-hexane, then filtered by a 0.45 μm organic filter. The following parameters were used: mobile phase = *n*-hexane/methanol/isopropanol (92.5/7.4/0.1, *v*/*v*/*v*), flow rate = 1.0 mL/min, column temperature = 30 °C, injection volume = 20.0 μL, and determining wavelength = 294 nm. Tocopherol and tocotrienol of MOs were identified and quantified by the retention time and standard curve.

### 2.6. Phytosterols Analysis

Phytosterols analysis were analyzed using a GC system equipped with a DB-5MS capillary column (0.25 μm, 30.0 m × 0.25 mm) and a FID (Agilent 7890A, Agilent Technologies, USA). First of all, 250 mg of MO was mixed with 1.0 mL of 5α-cholestane (0.5 mg/mL) and 3.0 mL of KOH-methanol (2.0 mol/L). The mixture was saponified at 85 °C for 1 h, then cooled to room temperature. 5.0 mL of *n*-hexane and 2.0 mL of distilled water were added to extract the phytosterols and repeated three times. The extract was dried by nitrogen and then silylated using 200 μL silylation reagents (pyridine/hexamethyldisilane/trimethylchlorosilane, 9/3/1, *v*/*v*/*v*) at 75 °C for 30 min. Then the silylated samples was cooled to room temperature and filtered by 0.22 μm organic membrane. After that, the filtered sample was determined using the following parameters: flow rate of helium = 3.0 mL/min; the initial temperature of the column = 230 °C, temperature programming to 280 °C = 10 °C/min, continued temperature programming to 290 °C = 5 °C/min, then held for 35 min, injection volume = 1.0 μL, injector temperature = 290 °C, and detector temperature = 300 °C. Phytosterols were identified and quantified by the retention time and internal standard substance.

### 2.7. Squalene Analysis

Squalene contents were determined using a GC system equipped with a HP-5 capillary column (0.25 μm, 30 m × 0.32 mm) and a FID (Agilent 7890A, Agilent Technologies, USA). A total 1.0 g of MO was mixed with 0.3 mL of squalane standard (1.0 mg/mL) and 50.0 mL of KOH-ethanol (1.0 mol/L). The mixture was saponified at 85 °C for 1.0 h. Then, distilled water (50.0 mL) was added to remove some water-soluble component, and cooled to room temperature. Then, 50.0 mL of *n*-hexane was added to extract the squalene and repeated three times. The combined *n*-hexane extracts were washed to pH 7.0 using 10% ethanol solution. The *n*-hexane was removed by rotary evaporator (Hei-vap Precision, Heidolph Co., Germany) at 30 °C to obtain the squalene samples. Finally, the squalene samples were dissolved in *n*-hexane again and reached to 10.0 mL. Then the sample filtered by 0.22 μm was determined. The following parameters were then used for measurement: flow rate of nitrogen = 1.0 mL/min; the initial temperature of the column = 160 °C, temperature programming to 220 °C = 15 °C/min, continued temperature programming to 280 °C = 5 °C/min (held for 20 min), continued temperature programming to 300 °C = 5 °C/min (held for 2 min), injection volume = 1.0 μL, injector temperature = 250 °C, and detector temperature = 300 °C. Squalene were identified and quantified by comparing the standards and internal standard of squalane.

### 2.8. The Total Polyphenol Content Analysis

The total polyphenol content was measured using the Folin–Ciocalteu reagent method based on the procedure of Gao, et al. [15] with some modifications. In brief, 4.0 g of MO was mixed with 3.0 mL of methanol using a vortex mixer (VX200-T, MET, USA) and placed in the dark to extract the total polyphenol. The extract processing was repeated three times, and the supernatants were combined. The supernatant (0.2 mL) was mixed with 0.8 mL of distilled water and 1.0 mL of Folin-Ciocalteu reagent and incubated for 5 min at room temperature. Then, 1.0 mL of sodium carbonate (7.5%) were added and incubated in the dark for 1.5 h. Afterwards, the absorbance was determined by UV-vis spectrophotometer (UV1700; Rangqi, Shanghai, China) at 760 nm. The results were expressed as mg of gallic acid equivalents (GAE) per kg of MO (mg/kg).

### 2.9. Mineral Composition Analysis

The mineral composition of the MO was detected according to the Inductively Coupled Plasma Mass Spectrometry (ICP-MS) method of Policarpi, et al. [16] with some modifications. 1.0 mL of hydrogen peroxide and 3.0 mL of nitric acid were added to 0.5 g of MO in closed microwave digestion tank. Then the digestion was according to the following procedure: microwave power was 1500 W, temperature programming from 0 to 120 °C within 5 min (held for 5 min), continued temperature programming to 200 °C within 5 min (held for 30 min). A total of 0.1 mg/L of arsenic was added as an internal standard substance. A multi-element stock standard solution containing all the analytical mineral elements was used to prepare a standard curve. The digested MO were dissolved in deionized water and analyzed using ICP-MS (Agilent 7900, Agilent Technologies, USA).

### 2.10. Determination of the Antioxidant Activity

Three different antioxidant models (DPPH, ABTS, and Ferric reducing ability of plasma (FRAP)) were employed to evaluate the antioxidant activity of MO (methanol extract mentioned above in Section 2.8) according to previous reports [15,17,18]. The antioxidant capacity of MO was expressed as Vitamin E equivalent (V_E_, μmol/kg).

### 2.11. Statistical Analysis

All the experiments were repeated in triplicate, and the date were expressed as means ± standard deviations. Statistical analysis was performed on SPSS 25.0 (SPSS Inc., Chicago, IL, USA). A comparison of the means was performed by Tukey’s test using one-way analysis of variance. MLR analysis using a stepwise method was performed to understand the correlations between antioxidant capacity assays and bioactive components. The use probability of F values *p*-to-enter and *p*-to-remove new variables into statistical model were *p* < 0.05 and *p* > 0.10, respectively.

## 3. Results and Discussion

### 3.1. Compositions of Macadamia Kernels

The compositions (lipid, crude protein, ash, and moisture content) of fifteen different cultivars of macadamia kernels were shown in Table 1. The protein content of macadamia kernels was averagely 8.07% with the highest value of 9.04% (Fuji (791)). The lipid contents of macadamia kernel ranged from 73.55% to 78.56% and the average was 75.98%. The GUANG 11 (14) contained the highest amount of oil compared to other varieties. The oil content in Chinese macadamia kernels was similar to that in New Zealand (69.1–78.4%) [7], but higher than that in Brazil (33.13% to 68.48%) [5,6]. Compared to some common edible nut seeds such as almond (~43.36%), cashew nut (~43.71%), walnut (~64.50%) [19], the macadamia kernels have a higher oil content, which can be a great potential oil sources in food industry. However, to be a good vegetable oil, it also needs to have the appropriate fatty acid composition, minor components, etc. Therefore, these components were analyzed in the next sections.

### 3.2. Fatty Acid and Triacylglycerol

#### 3.2.1. Fatty Acid Composition and Content

The fatty acid composition is an important parameter of the quality and authenticity of oil, which can be used to detect the frauds of oil. Fatty acid composition and content of 15 different MO were shown in Table 2. The oil samples contained twelve fatty acids, and the major fatty acids were unsaturated fatty acids (UFAs). The sum of UFAs (including palmitoleic acid (C16:1), oleic acid (C18:1), linoleic acid (C18:2), linolenic acid (C18:3), eicosenoic acid (C20:1) and docosenoic acid (C22:1)) accounted for more than 83% of the total fatty acid content, where Fuji (791) (2) had the highest UFAs value (85.9%). In addition, monounsaturated fatty acids (MUFAs) accounted for 96.67–98.26% of UFAs, mainly consist of C18:1 (61.74–66.47% of fatty acid) and C16:1 (13.22–17.63% of fatty acid). The fatty acid composition was similar to previous reports [1,7,19,20,21]. However, the contents were different, which might relate to the cultivar, kernel maturity, geographical location and growth conditions. It was reported that diets containing high oleic acid and MUFA contents reduce low-density lipoprotein levels and decrease the risk of cardiovascular diseases. Moreover, vegetable oils with high oleic acid content are highly desirable in terms of thermal stability and longer shelf life [22,23]. Therefore, MO has potential as a healthy vegetable oil.

#### 3.2.2. Triacylglycerol Composition

The triacylglycerol composition in the oil is also an important index of quality which is widely used in the industry to control the purity of oil and identify authenticity [24]. However, the composition and content of triacylglycerol of MO has not been yet reported elsewhere, which were analyzed in this study and presented in Table 3. Twenty kinds of triacylglycerols in MO were identified and quantified, namely 1,3-dimyristoyl-2-oleoyl-glycerol (MOM), 1,2-dipalmitoyl-3-palmitoleyl-glycerol (PPP_O_), 1-myristoyl-2-oleoyl-3-palmitoyl-glycerol (MOP), 1-myristoyl-2-linoleoyl-3-palmitoyl-glycerol (MLP), 1,3-dipalmitoyl-2-oleyl-glycerol (POP), 1-myristoyl-2,3-dioleoyl-glycerol (MOO), 1-palmitoyl-2-linoleoyl-3-palmitoleyl-glycerol (PLP_O_), 1-palmitoyl-2,3-distearoyl-glycerol (PSS), 1-palmitoleyl-2-oleyl-3-stearoyl-glycerol (P_O_OS), 1-palmitoyl-2,3-dioleoyl-glycerol (POO), 1-palmitoleyl-2-linoleoyl-3-stearoyl-glycerol (P_O_LS), 1-palmitoyl-2-linoleoyl-3-oleyl-glycerol (PLO), 1-palmitoyl-2,3-dilinoleoyl-glycerol (PLL), 1-stearoyl-2-linoleoyl-3-oleyl-glycerol (SLO), 1,3-dioleoyl-2-linoleoyl-glycerol (OLO), 1-stearoyl-2-oleyl-3-arachidyl-glycerol (SOA), 1-arachidyl-2,3-dioleyl-glycerol (AOO), 1,3-distearoyl-2-oleyl-glycerol (SOS), 1-stearoyl-2,3-dioleyl-glycerol (SOO), and triolein (OOO). The main triacylglycerol included OOO (19.18–26.14%), POO (16.36–18.19%), P_O_OS (11.87–13.65%), POP (6.89–8.96%), MOO (6.08–8.46%) and SOO (4.81–6.93%), which accounted for more than 70% of total triacylglycerol. In addition, as shown in Table 3, the triacylglycerol contents also had a certain difference among cultivars. The OOO of the Fuji (791) (2) had the highest value (26.14%).

Both fatty acid and triacylglycerol compositions indicated that MO is a good source of UFAs. According to the Standards issued by the Food and Agriculture Organization of the United Nations, the MUFAs content of healthy edible oils should be higher than 75% [25]. Thus, MO has potential as a dietary resource of plant oil.

### 3.3. Minor Components

The minor components including polyphenols, tocopherols, squalene, minerals and phytosterols of MO from different cultivars were determined, which were important quality and nutritional characteristics of vegetable oil [26,27].

#### 3.3.1. Polyphenols Content

Polyphenols are important minor components in vegetable oils because they confer the sensory and nutritional characteristics [28]. The content of polyphenol in MOs was shown in Table 4. In general, the content of polyphenols from different cultivars showed large differences. Among the 15 cultivars, the polyphenols contents ranged from 19.74 to 123.40 GAE mg/kg. The polyphenols content of Fuji (791) (2) was about 6 times higher than that of Hinde (H2) (1). Quinn and Tang [29] reported the polyphenols content of MO from Hawaii was 48.7 GAE mg/kg, which is within the scope of our results. However, the polyphenol contents of most cultivars in our study were higher than the report of Cicero, et al. [30], who indicated the polyphenols content of MO from Brazilian was only 2.36 GAE mg/kg. These differences may be related to the macadamia growing conditions, variety and location.

#### 3.3.2. Tocopherol Content

The profile of tocopherols in 15 Chinese macadamia cultivars was shown in Table 4. It was worth noting that only α-tocotrienol was identified in the MO and the content ranged from 27.9 to 53.1 mg/kg. The highest content of α-tocotrienols was observed in the A4 (9), while the lowest content in HAES 863 (8). The profile of tocopherols may be related to the planting area and cultivars. For example, in Hawaii, the total tocopherol content of MO was 9–25 mg/g [28], while no tocopherol was detected in New Zealand [7]. In addition, seven different macadamia cultivars from the island of Hawaii were determined, and α-tocotrienol was 15.91–46.83 mg/g, ε-tocotrienol ranged from 3.00–17.66 mg/g, and γ-tocotrienol was 8.75–34.28 mg/g of oil, whereas the α- and γ-tocopherol compounds were only detected in two cultivars [10]. Compared to the tocotrienols contents of 21 species of plant oils reported by Gruszka and Kruk [31], the MOs in our study had higher content of α-tocotrienol. As we all know, tocotrienols are more powerful antioxidants than tocopherols, and showed better cholesterol-lowering and anti-cancer properties [10,32]. In addition, it can quickly penetrate the skin and reduce oxidative stress caused by ultraviolet light [10]. Therefore, MO may be used in functional foods and cosmetics.

#### 3.3.3. Squalene Content

Squalene is a triterpene precursor to the biosynthesis of vitamin D, steroid hormones, and cholesterol in the human body, which is beneficial to health [33]. As shown in Table 4, the squalene contents of MO ranged from 91.15 to 268.08 mg/kg. The highest content of squalene was observed in the Fuji (791) (2), while the lowest content in the Hinde (H2) (1). The results were in accordance with [10,19], who reported the contents of MO ranged from 72–185 mg/kg, but significantly higher than cold-pressed MO (22.9 mg/kg), which was sold in the market of Brazilian [29]. In addition, the squalene content of MO was relatively higher than nut oils such as hazelnuts (186.4 mg/kg), peanuts (98.3 mg/kg), almonds (95.0 mg/kg), and walnuts (9.4 mg/kg) [20]. Squalene, like tocotrienol, is a potent and stable antioxidant [10]. In addition, research indicated that long-term consumption of squalene-rich vegetable oil can inhibit forming tumors and less often develop various cancers [34]. Therefore, MO was a potential source of dietary squalene.

#### 3.3.4. Phytosterols Content

Phytosterols, which was usually considered as a nutritional supplement for fats and oils, and was one of the important bioactive classes of constitutes in nut oils [35]. The profile of phytosterols in 15 Chinese macadamia varieties was shown in Table 5. Eight kinds of phytosterols were identified and quantified, namely β-sitosterol (1248.8 to 1613.7 mg/kg), Δ^5^-avenasterol (170.0 to 362.1 mg/kg), campesterol (82.4 to 114.1 mg/kg), Δ^7^-stigmastenol (7.8 to 48.6 mg/kg), Δ^7^-avenasterol (7.6 to 41.3 mg/kg), clerosterol (11.4 to 16.3 mg/kg), Δ^5,24^-stigmastadienol (7.8 to 19.8 mg/kg), β-stiostanol (5.4 to 9.6 mg/kg). The total content of phytosterols ranged from 1569.50 to 2064.54 mg/kg. Among the identified phytosterols, β-sitosterol, Δ^5^-avenasterol and campesterol were the major, which accounted for more than 92% of the total phytosterols. Furthermore, β-sitosterol content was the highest, which was similar to previous reports [9,36]. It was worth noting that phytosterol content in MO from different cultivars had certain differences but not significant. Thus, the detailed phytosterol profile can be used as a fingerprint to identify the vegetable oils.

#### 3.3.5. Mineral Content

The composition and content of minerals also affects the quality of some oils. Although minerals in macadamia nuts/kernel have been widely reported, those in MO is very rare, especially among different varieties. Seven kinds of minerals (Mg, Ca, Zn, As, Pb, K and Na) were determined, but only four kinds of minerals (Mg, Ca, K and Na) were identified and quantified, ranged from 8.13 to 49.90, 0 to 28.50, 13.75 to 51.80 and 0.41 to 2.51 mg/kg, respectively (Table 4). These minerals in oil were beneficial to the health of adults as suggested by dietary recommended intake guidelines. For example, Ca is associated with bone health and prevention of osteoporosis, and Mg is associated with activation of enzymatic systems [37]. Moreover, Aslanabadi, et al. [38] reported that long-term consumption of natural mineral foods rich in Ca, Mg, and bicarbonate can reduce cholesterol and low density lipoprotein. Hence, the relatively high minerals content can promote the quality and nutrition of MO.

### 3.4. In Vitro Antioxidant Capacity

The antioxidant capacity of MOs determined using different models was shown in Table 4. The antioxidant capacity analyzed by DPPH, ABTS and FRAP model was 126–359, 136–324, and 255–1086 μmol V_E_/kg, respectively. Among the three models, the antioxidant activity had the same tendency, but presented different absolute values. The value difference may be due to the different chemical mechanisms of the antioxidant models [39,40]. Fuji (791) (2) showed the highest antioxidant capacity, while the Hinde (H2) (1) exhibited the lowest. It was noted that the study only analyzed the characteristics of different varieties of MO in one region for one year, but the growth conditions had a great influence on the composition and content of minor components. In order to obtain more detailed information, the team will continue to conduct long-term monitoring of MO from different regions, years and varieties in China. According to the references, the differences in antioxidant capacity of materias may be contributed to polyphenols, squalene, phytosterols, etc. [15,17,18,39]. Therefore, it is necessary to clarify the relationship between antioxidant capacities and minor compounds.

### 3.5. Correlations between Antioxidant Capacity and Minor Components

Bivariate correlations analysis as a common method was utilized to investigate the correlation between two continuous variables and calculate the correlation coefficient (r) [15,17]. The correlation between antioxidant capacities and minor compounds was shown in Table 6. The antioxidant capacity showed a significant positive correlation with the polyphenols (r was 0.938–0.965, *p* < 0.01) and squalene content (r was 0.870–0.927, *p* < 0.01). This result was similar with previous studies. Gao, et al. [17] reported that the oxidative stability index of walnut oil was significantly correlated with polyphenols (r = 0.873, *p* < 0.01), squalene (r = 0.701, *p* < 0.01), and stigmasterol (r = 0.770, *p* < 0.01). Shi, et al. [41] also reported that remarkable positive correlations were observed between squalene and measured by DPPH, ABTS and FRAP (r was 0.82–0.91), and the correlations between polyphenol and antioxidant capacity were also positive (r was 0.65–0.67). The analysis strongly supported the positive contribution of polyphenols and squalene to the antioxidant capacity of MOs.

### 3.6. MLR Analysis

MLR analysis can be considered as a useful tool to analyze a phenomenon (antioxidant capacity) associated with multiple factors (minor components) [15,17]. In this study, a regression model was defined: Y as the dependent variable and X as independent variables (minor components). Linear models were constructed in the form as: Y = M_0_ + M_1_X_1_ + M_2_X_2_ + M_3_X_3_ + … + M_n_X_n_.

Where, Y represents the predicted response, M_0_ denotes the unstandardized constant coefficient, M_1_, M_2_, M_3_ and M_n_ refer to partial correlation coefficients, and X_1_, X_2_, X_3_ and X_n_ represent the independent variables [15,17].

Based on the analysis of a stepwise method of MLR, polyphenols and squalene exhibited a good correlation. As shown in Table 7, the DPPH-based model showed the highest value of adjusted R^2^ (0.937), with the constant of the unstandardized coefficients was 35.671, and the partial correlation coefficients (R) of the predicted equation was 0.546 and 0.467 for polyphenols and squalene, respectively (Y = 35.671 + 0.546 polyphenols + 0.467 squalene). Moreover, polyphenols (R = 0.621) and squalene (R = 0.376) also affected ABTS radical scavenging activity and polyphenols (R = 0.965) influenced FRAP reducing capacity. In accordance with the correlation results mentioned in Section 3.5, polyphenols (r was 0.938–0.965) and squalene (r was 0.870–0.927) showed high regression coefficients and significant correlations (*p* < 0.01) in all antioxidant model. Taking together, the polyphenols and squalene were considered as an indicator of the antioxidant capacity of the MO. This result can provide a valuable judge for botanists and consumers.

## 4. Conclusions

The chemical compositions and antioxidant capacities of oils obtained from 15 macadamia cultivars grown in China were analyzed. Generally, the macadamia kernel is rich in oils (average of 75.98%). The major fatty acids in MOs were monounsaturated fatty acids, mainly including oleic acid and palmitoleic acid. The main triacylglycerols were OOO, POO and P_O_OS, which was first time reported for MO. Furthermore, MO contained high minor components including α-tocotrienols, phytosterols, squalene, and polyphenols. Among all the cultivars, Fuji (791) (2) contained the highest polyphenols (123.4 mg/kg), squalene (268.1 mg/kg) and exhibited the highest antioxidant capacity. As correlation analysis indicated, polyphenols and squalene content positively correlated with the antioxidant capacity of MOs. In addition, the antioxidant capacity can be predicted by the model of MLR. The above results indicated that MO has potential as a healthy vegetable oil and offer a valuable directive for the breeding of macadamia in China.

## Figures and Tables

**Table 1:** Chemical composition of the macadamia kernels ^1^.

No.	Moisture Content	Ash Content	Protein Content	Oil Content
1	1.62 ± 0.03 ^a^	1.25 ± 0.01 ^b^	8.55 ± 0.08 ^c^	75.02 ± 0.11 ^b^
2	1.65 ± 0.06 ^a,b^	1.13 ± 0.07 ^a^	9.04 ± 0.13 ^d,e^	76.11 ± 0.22 ^e^
3	1.55 ± 0.11 ^a^	1.21 ± 0.04 ^a,b^	7.88 ± 0.16 ^b^	75.45 ± 0.19 ^b,c^
4	1.63 ± 0.00 ^a^	1.16 ± 0.05 ^a^	8.21 ± 0.16 ^c^	77.19 ± 0.04 ^f^
5	1.74 ± 0.01 ^c^	1.33 ± 0.01 ^c^	8.64 ± 0.13 ^c,d^	73.55 ± 0.06 ^a^
6	1.66 ± 0.10 ^a^	1.43 ± 0.09 ^c,d^	7.23 ± 0.23 ^a,b^	77.32 ± 0.05 ^c^
7	1.70 ± 0.05 ^a,b,c^	1.36 ± 0.03 ^c^	7.83 ± 0.14 ^b^	75.71 ± 0.22 ^b,d^
8	1.86 ± 0.16 ^b,c^	1.14 ± 0.06 ^a^	8.83 ± 0.07 ^d^	75.37 ± 0.06 ^c^
9	1.70 ± 0.03 ^b^	1.48 ± 0.03 ^d^	7.74 ± 0.10 ^b^	76.33 ± 0.21 ^e^
10	1.68 ± 0.06 ^a,b,c^	1.39 ± 0.02 ^c^	7.66 ± 0.08 ^a,b^	76.16 ± 0.25 ^e^
11	1.65 ± 0.02 ^a^	1.47 ± 0.03 ^d^	7.55 ± 0.10 ^b^	75.94 ± 0.15 ^e^
12	1.66 ± 0.10 ^a^	1.24 ± 0.10 ^b^	7.17 ± 0.25 ^a^	75.32 ± 0.23 ^b,c^
13	1.73 ± 0.11 ^a,b,c^	1.28 ± 0.05 ^b,c^	7.94 ± 0.16 ^b,c^	75.77 ± 0.01 ^d,e^
14	1.71 ± 0.03 ^b,c^	1.17 ± 0.01 ^a^	8.94 ± 0.26 ^c,d,e^	78.36 ± 0.36 ^g^
15	1.63 ± 0.01 ^a^	1.26 ± 0.02 ^b^	7.78 ± 0.07 ^b^	76.04 ± 0.33 ^e^
Max	1.86 ± 0.16 ^b,c^	1.48 ± 0.03 ^d^	9.04 ± 0.13 ^d,e^	78.36 ± 0.36 ^g^
Min	1.55 ± 0.11 ^a^	1.13 ± 0.07 ^a^	7.17 ± 0.25 ^a^	73.55 ± 0.06 ^a^
Average	1.68 ± 0.05 ^a,b,c^	1.29 ± 0.04 ^b,c^	8.07 ± 0.14 ^b,c^	75.98 ± 0.13 ^e^

^1^ Values are means ± SD. Different letters in a column indicate significant differences (*p* < 0.05).

**Table 2:** Fatty acid compositions (%) of oils extracted from 15 macadamia cultivars ^1^.

No.	C14:0 *	C16:0	C16:1	C18:0	C18:1	C18:2	C18:3	C20:0	C20:1	C22:0	C22:1	C24:0	SFA	MUFA	PUFA	UFA
1	0.72 ± 0.02 ^j^	8.22 ± 0.00 ^k^	16.44 ± 0.01 ^j^	3.24 ± 0.00 ^e,f^	63.22 ± 0.04 ^c^	1.84 ± 0.01 ^e^	0.21 ± 0.00 ^b^	2.54 ± 0.00 ^a^	2.25 ± 0.00 ^b^	0.76 ± 0.02 ^a^	0.19 ± 0.00 ^a^	0.36 ± 0.03 ^a,b^	15.84 ± 0.02 ^g^	82.10 ± 0.01 ^e^	2.05 ± 0.00 ^c^	84.15 ± 0.00 ^e^
2	0.52 ± 0.01 ^g^	6.81 ± 0.01 ^a^	13.73 ± 0.04 ^c^	2.61 ± 0.01 ^b^	66.47 ± 0.01 ^l^	2.07 ± 0.00 ^h^	0.26 ± 0.00 ^e^	2.75 ± 0.00 ^e^	3.04 ± 0.02 ^m^	0.99 ± 0.00 ^f^	0.33 ± 0.02 ^g^	0.42 ± 0.00 ^d^	14.10 ± 0.01 ^a^	83.57 ± 0.02 ^l^	2.33 ± 0.00 ^d^	85.90 ± 0.01 ^l^
3	0.50 ± 0.00 ^f^	7.45 ± 0.01 ^f^	15.64 ± 0.01 ^k^	3.22 ± 0.00 ^e^	64.79 ± 0.02 ^g^	1.16 ± 0.00 ^a^	0.31 ± 0.00 ^h^	2.79 ± 0.02 ^f^	2.55 ± 0.00 ^h^	0.92 ± 0.00 ^d^	0.27 ± 0.00 ^e^	0.39 ± 0.00 ^b^	15.27 ± 0.00 ^d^	83.25 ± 0.01 ^k^	1.47 ± 0.00 ^a^	84.72 ± 0.00 ^i^
4	0.51 ± 0.03 ^f,g^	7.81 ± 0.00 ^h^	14.62 ± 0.00 ^g^	3.71 ± 0.03 ^l^	65.17 ± 0.02 ^i^	1.47 ± 0.00 ^c^	0.20 ± 0.00 ^a^	2.94 ± 0.00 ^h^	2.22 ± 0.00 ^a^	0.82 ± 0.03 ^b^	0.18 ± 0.03 ^a^	0.36 ± 0.00 ^a^	16.15 ± 0.03 ^j^	82.19 ± 0.01 ^e^	1.67 ± 0.00 ^b^	83.86 ± 0.00 ^c^
5	0.50 ± 0.00 ^f^	8.24 ± 0.02 ^k^	17.11 ± 0.01 ^l^	3.99 ± 0.00 ^n^	61.74 ± 0.04 ^a^	1.17 ± 0.00 ^a^	0.28 ± 0.00 ^f^	3.14 ± 0.01 ^k^	2.29 ± 0.00 ^c^	0.92 ± 0.01 ^d^	0.21 ± 0.00 ^b^	0.41 ± 0.01 ^c^	17.20 ± 0.01 ^l^	81.35 ± 0.01 ^c^	1.45 ± 0.00 ^a^	82.80 ± 0.00 ^a^
6	0.40 ± 0.01 ^c^	7.62 ± 0.00 ^g^	13.22 ± 0.02 ^a^	3.85 ± 0.00 ^m^	64.71 ± 0.10 ^f^	2.53 ± 0.01 ^j^	0.26 ± 0.01 ^e^	3.01 ± 0.00 ^j^	2.78 ± 0.01 ^k^	0.92 ± 0.00 ^d^	0.29 ± 0.00 ^f^	0.41 ± 0.00 ^c^	16.21 ± 0.00 ^j^	81.00 ± 0.00 ^a^	2.79 ± 0.00 ^f^	83.79 ± 0.00 ^c^
7	0.43 ± 0.00 ^d^	7.80 ± 0.01 ^h^	14.64 ± 0.00 ^g^	3.58 ± 0.00 ^k^	64.94 ± 0.03 ^h^	1.44 ± 0.01 ^c^	0.25 ± 0.01 ^e^	2.97 ± 0.01 ^i^	2.42 ± 0.01 ^f^	0.82 ± 0.00 ^b^	0.22 ± 0.00 ^c^	0.42 ± 0.01 ^c,d^	16.02 ± 0.00 ^i^	82.22 ± 0.02 ^f^	1.69 ± 0.00 ^b^	83.91 ± 0.01 ^d^
8	0.46 ± 0.01 ^e^	7.94 ± 0.03 ^i^	14.46 ± 0.00 ^f^	3.18 ± 0.01 ^d^	64.63 ± 0.00 ^f^	2.02 ± 0.00 ^g^	0.23 ± 0.00 ^d^	2.73 ± 0.01 ^d^	2.76 ± 0.00 ^j^	0.89 ± 0.02 ^c^	0.29 ± 0.01 ^f^	0.42 ± 0.00 ^c,d^	15.62 ± 0.02 ^f^	82.14 ± 0.00 ^e^	2.25 ± 0.00 ^d^	84.39 ± 0.00 ^g^
9	0.30 ± 0.01 ^a^	8.29 ± 0.02 ^l^	15.16 ± 0.03 ^i^	3.41 ± 0.01 ^i^	63.98 ± 0.00 ^d^	1.86 ± 0.00 ^e^	0.22 ± 0.00 ^c^	2.91 ± 0.01 ^f^	2.34 ± 0.00 ^d^	0.91 ± 0.00 ^c,d^	0.21 ± 0.00 ^b^	0.41 ± 0.00 ^c^	15.96 ± 0.01 ^h^	81.69 ± 0.01 ^d^	2.08 ± 0.00 ^c^	83.77 ± 0.00 ^c^
10	0.34 ± 0.00 ^b^	7.37 ± 0.00 ^d^	13.57 ± 0.01 ^b^	4.08 ± 0.00 ^o^	64.93 ± 0.01 ^h^	2.17 ± 0.00 ^i^	0.22 ± 0.01 ^b,c^	3.17 ± 0.00 ^l^	2.49 ± 0.00 ^g^	0.97 ± 0.02 ^e,f^	0.25 ± 0.00 ^d^	0.44 ± 0.03 ^c,d,e^	16.37 ± 0.01 ^k^	81.24 ± 0.00 ^b^	2.39 ± 0.00 ^e^	83.63 ± 0.00 ^b^
11	0.63 ± 0.00 ^i^	7.25 ± 0.00 ^c^	14.06 ± 0.00 ^d^	2.69 ± 0.03 ^c^	65.55 ± 0.01 ^k^	1.98 ± 0.00 ^f^	0.29 ± 0.00 ^g^	2.63 ± 0.00 ^c^	3.13 ± 0.02 ^n^	0.94 ± 0.01 ^e^	0.40 ± 0.03 ^h^	0.44 ± 0.00 ^e^	14.58 ± 0.01 ^c^	83.14 ± 0.02 ^j^	2.27 ± 0.00 ^d^	85.41 ± 0.01 ^j^
12	0.81 ± 0.02 ^k^	6.98 ± 0.01 ^b^	14.89 ± 0.01 ^h^	3.55 ± 0.01 ^j^	65.47 ± 0.02 ^k^	1.37 ± 0.01 ^b^	0.27 ± 0.01 ^e,f^	2.91 ± 0.00 ^f^	2.36 ± 0.00 ^e^	0.83 ± 0.01 ^b^	0.21 ± 0.01 ^b^	0.34 ± 0.03 ^a^	15.42 ± 0.02 ^e^	82.93 ± 0.02 ^i^	1.64 ± 0.01 ^b^	84.57 ± 0.02 ^h^
13	0.55 ± 0.01 ^h^	7.40 ± 0.00 ^e^	14.70 ± 0.02 ^g^	2.54 ± 0.00 ^a^	65.26 ± 0.00 ^j^	1.75 ± 0.01 ^d^	0.31 ± 0.01 ^h^	2.56 ± 0.00 ^b^	3.21 ± 0.01 ^o^	0.92 ± 0.00 ^d^	0.38 ± 0.00 ^h^	0.44 ± 0.02 ^d,e^	14.41 ± 0.01 ^b^	83.55 ± 0.01 ^l^	2.06 ± 0.01 ^c^	85.61 ± 0.01 ^k^
14	0.82 ± 0.00 ^k^	7.47 ± 0.00 ^f^	14.27 ± 0.00 ^e^	3.31 ± 0.00 ^g^	64.23 ± 0.01 ^e^	1.99 ± 0.00 ^f^	0.29 ± 0.00 ^g^	3.02 ± 0.01 ^j^	2.84 ± 0.01 ^l^	1.05 ± 0.00 ^g^	0.31 ± 0.00 ^g^	0.39 ± 0.00 ^b^	16.06 ± 0.00 ^i^	81.65 ± 0.01 ^d^	2.28 ± 0.00 ^d^	83.93 ± 0.00 ^d^
15	0.42 ± 0.01 ^c,d^	8.00 ± 0.02 ^j^	17.63 ± 0.01 ^m^	3.25 ± 0.00 ^f^	62.51 ± 0.00 ^b^	1.37 ± 0.00 ^b^	0.36 ± 0.01 ^i^	2.76 ± 0.01 ^e,f^	2.31 ± 0.00 ^d^	0.82 ± 0.00 ^b^	0.22 ± 0.01 ^b,c^	0.36 ± 0.01 ^a^	15.61 ± 0.01 ^f^	82.67 ± 0.01 ^h^	1.73 ± 0.00 ^b^	84.40 ± 0.00 ^g^
Max	0.82 ± 0.00 ^k^	8.29 ± 0.02 ^l^	17.63 ± 0.01 ^m^	4.08 ± 0.00 ^o^	66.47 ± 0.01 ^l^	2.53 ± 0.01 ^j^	0.36 ± 0.01 ^i^	3.17 ± 0.00 ^k^	3.21 ± 0.01 ^n^	1.05 ± 0.00 ^g^	0.40 ± 0.03 ^h^	0.44 ± 0.00 ^e^	17.20 ± 0.01 ^l^	83.57 ± 0.02 ^l^	2.79 ± 0.00 ^f^	85.90 ± 0.01 ^l^
Min	0.30 ± 0.01 ^a^	6.81 ± 0.01 ^a^	13.22 ± 0.02 ^a^	2.54 ± 0.00 ^a^	61.74 ± 0.04 ^a^	1.16 ± 0.00 ^a^	0.20 ± 0.00 ^a^	2.54 ± 0.00 ^a^	2.22 ± 0.00 ^a^	0.76 ± 0.02 ^a^	0.18 ± 0.03 ^a^	0.36 ± 0.00 ^a^	14.10 ± 0.01 ^a^	81.00 ± 0.00 ^a^	1.45 ± 0.00 ^a^	82.80 ± 0.00 ^a^

^1^ Values are means ± SD. Different letters in a column indicate significant differences (*p* < 0.05). * C14:0, myristic acid; C16:0, palmitic acid; C16:1, palmitoleic acid; C18:0, stearic acid; C18:1, oleic acid; C18:2, linoleic acid; C18:3, linolenic acid; C20:0, eicosanoic acid; C20:1, eicosenoic acid; C22:0, docosanoic acid; C22:1, docosenoic acid; C24:0, lignoceric acid. SFA, saturated fatty acids (C14:0 + C16:0 + C18:0 + C20:0 + C22:0 + C24:0); MUFA, mono-unsaturated fatty acids (C16:1 + C18:1 + C20:1 + C22:1); PUFA, poly-unsaturated fatty acids (C18:2+ C18:3); UFA, unsaturated fatty acids (MUFA + PUFA).

**Table 3:** Triacylglycerols [%(w/w)] of oils extracted from 15 macadamia cultivars ^1^.

No.	1	2	3	4	5	6	7	8	9	10	11	12	13	14	15
MOM *	0.25 ± 0.00 ^f^	0.19 ± 0.00 ^d,e^	0.19 ± 0.00 ^d,e^	0.17 ± 0.00 ^d^	0.20 ± 0.00 ^e^	0.11 ± 0.00 ^a^	0.13 ± 0.01 ^b^	0.15 ± 0.00 ^c^	0.10 ± 0.00 ^a^	0.11 ± 0.00 ^a^	0.20 ± 0.00 ^e^	0.28 ± 0.01 ^g^	0.19 ± 0.00 ^d,e^	0.26 ± 0.00 ^f^	0.17 ± 0.01 ^c,d^
PPP_O_	0.81 ± 0.02 ^h^	0.51 ± 0.00 ^b^	0.60 ± 0.00 ^e^	0.61 ± 0.00 ^e^	0.72 ± 0.01 ^g^	0.52 ± 0.00 ^b^	0.57 ± 0.00 ^d^	0.60 ± 0.00 ^e^	0.56 ± 0.01 ^c,d^	0.47 ± 0.00 ^a^	0.56 ± 0.00 ^c^	0.63 ± 0.00 ^f^	0.55 ± 0.00 ^c^	0.70 ± 0.00 ^g^	0.60 ± 0.00 ^e^
MOP	2.26 ± 0.01 ^k^	1.63 ± 0.00 ^d^	1.87 ± 0.00 ^g^	1.75 ± 0.00 ^f^	2.16 ± 0.01 ^j^	1.44 ± 0.00 ^b^	1.59 ± 0.01 ^c,d^	1.61 ± 0.01 ^d^	1.59 ± 0.00 ^c^	1.38 ± 0.01 ^a^	1.65 ± 0.00 ^d^	1.94 ± 0.01 ^h^	1.70 ± 0.00 ^e^	1.94 ± 0.00 ^h^	1.98 ± 0.00 ^i^
MLP	1.28 ± 0.01 ^h^	0.96 ± 0.00 ^c^	1.15 ± 0.00 ^g^	1.07 ± 0.01 ^e^	1.41 ± 0.00 ^i^	0.88 ± 0.00 ^a^	0.95 ± 0.00 ^c^	0.88 ± 0.01 ^a^	1.12 ± 0.00 ^f^	0.92 ± 0.00 ^b^	0.89 ± 0.00 ^a^	0.99 ± 0.01 ^d^	1.09 ± 0.00 ^e^	0.95 ± 0.00 ^c^	1.64 ± 0.00 ^j^
PLP_O_	2.18 ± 0.00 ^h^	1.54 ± 0.02 ^a^	1.84 ± 0.01 ^d^	2.06 ± 0.00 ^g^	2.28 ± 0.02 ^i^	1.94 ± 0.02 ^e^	2.03 ± 0.00 ^f^	2.07 ± 0.02 ^g^	2.23 ± 0.02 ^i^	1.88 ± 0.02 ^d^	1.61 ± 0.01 ^b^	1.63 ± 0.01 ^b^	1.69 ± 0.01 ^c^	1.84 ± 0.01 ^d^	1.95 ± 0.00 ^e^
POP	8.96 ± 0.01 ^l^	6.94 ± 0.02 ^a^	8.00 ± 0.01 ^i^	7.87 ± 0.00 ^g^	8.89 ± 0.02 ^k^	7.00 ± 0.01 ^b^	7.77 ± 0.00 ^f^	7.88 ± 0.02 ^g^	7.91 ± 0.01 ^g,h^	6.89 ± 0.01 ^a^	7.01 ± 0.01 ^b^	7.46 ± 0.00 ^d^	7.15 ± 0.01 ^c^	7.62 ± 0.01 ^e^	8.07 ± 0.00 ^j^
MOO	7.98 ± 0.01 ^l^	6.35 ± 0.01 ^d^	7.49 ± 0.00 ^k^	6.96 ± 0.00 ^i^	8.24 ± 0.01 ^m^	6.08 ± 0.01 ^a^	6.81 ± 0.00 ^h^	6.68 ± 0.01 ^g^	7.23 ± 0.01 ^j^	6.23 ± 0.02 ^c^	6.13 ± 0.00 ^b^	6.61 ± 0.01 ^f^	6.70 ± 0.01 ^g^	6.24 ± 0.01 ^c^	8.46 ± 0.00 ^e^
PSS	1.50 ± 0.00 ^f^	1.10 ± 0.00 ^a,b^	1.33 ± 0.00 ^c^	1.60 ± 0.00 ^h^	1.85 ± 0.00 ^j^	1.61 ± 0.00 ^h^	1.55 ± 0.00 ^g^	1.41 ± 0.00 ^e^	1.59 ± 0.00 ^h^	1.67 ± 0.00 ^i^	1.12 ± 0.00 ^b^	1.41 ± 0.00 ^e^	1.08 ± 0.00 ^a^	1.40 ± 0.00 ^e^	1.36 ± 0.00 ^d^
P_O_OS	13.00 ± 0.01 ^g^	12.12 ± 0.00 ^b^	12.51 ± 0.01 ^c^	13.11 ± 0.00 ^h^	12.72 ± 0.01 ^f^	13.22 ± 0.01 ^j^	13.26 ± 0.00 ^k^	13.19 ± 0.01 ^i^	13.65 ± 0.01 ^l^	12.71 ± 0.01 ^f^	12.67 ± 0.00 ^e^	11.87 ± 0.00 ^a^	12.61 ± 0.00 ^d^	12.54 ± 0.01 ^c^	12.47 ± 0.00 ^c^
P_O_LS	2.38 ± 0.00 ^h^	2.01 ± 0.00 ^d^	2.34 ± 0.00 ^g^	2.61 ± 0.00 ^k^	2.87 ± 0.00 ^l^	2.00 ± 0.00 ^d^	2.45 ± 0.00 ^i^	2.31 ± 0.00 ^f^	2.32 ± 0.00 ^f^	2.54 ± 0.00 ^j^	1.54 ± 0.00 ^a^	2.21 ± 0.00 ^e^	1.60 ± 0.00 ^b^	1.95 ± 0.00 ^c^	2.21 ± 0.00 ^e^
POO	18.00 ± 0.02 ^i^	17.26 ± 0.00 ^e^	18.19 ± 0.01 ^j^	17.00 ± 0.00 ^d^	17.55 ± 0.01 ^g^	16.43 ± 0.01 ^b^	17.37 ± 0.00 ^f^	17.81 ± 0.01 ^h^	16.91 ± 0.01 ^c^	16.36 ± 0.01 ^a^	17.36 ± 0.00 ^f^	17.54 ± 0.01 ^g^	17.27 ± 0.00 ^e^	16.98 ± 0.01 ^d^	18.01 ± 0.00 ^i^
PLO	2.60 ± 0.00 ^i^	2.29 ± 0.00 ^d^	2.60 ± 0.00 ^i^	2.48 ± 0.00 ^g^	2.79 ± 0.00 ^k^	2.10 ± 0.00 ^b^	2.78 ± 0.00 ^k^	2.43 ± 0.00 ^e^	3.02 ± 0.00 ^l^	2.45 ± 0.00 ^f^	2.21 ± 0.00 ^c^	2.45 ± 0.00 ^f^	2.59 ± 0.00 ^h^	1.90 ± 0.00 ^a^	2.73 ± 0.00 ^j^
PLL	0.99 ± 0.00 ^h^	1.00 ± 0.00 ^h,i^	0.61 ± 0.00 ^a^	0.72 ± 0.00 ^c^	0.66 ± 0.00 ^b^	1.30 ± 0.00 ^k^	0.75 ± 0.00 ^d^	1.05 ± 0.00 ^j^	0.95 ± 0.00 ^f^	0.97 ± 0.00 ^g^	1.01 ± 0.00 ^i^	0.73 ± 0.00 ^c^	0.91 ± 0.00 ^e^	1.01 ± 0.00 ^i^	0.76 ± 0.00 ^d^
SOS	1.13 ± 0.00 ^c^	1.12 ± 0.00 ^c^	1.18 ± 0.00 ^d^	1.35 ± 0.00 ^h^	1.52 ± 0.00 ^k^	1.59 ± 0.00 ^m^	1.39 ± 0.00 ^j^	1.28 ± 0.00 ^g^	1.37 ± 0.00 ^i^	1.57 ± 0.00 ^l^	1.08 ± 0.00 ^b^	1.25 ± 0.00 ^f^	1.03 ± 0.00 ^a^	1.36 ± 0.00 ^h,i^	1.20 ± 0.00 ^e^
SOO	5.25 ± 0.00 ^c^	5.06 ± 0.00 ^b^	5.71 ± 0.01 ^h^	6.35 ± 0.00 ^l^	6.13 ± 0.00 ^j^	6.93 ± 0.00 ^m^	6.24 ± 0.00 ^k^	5.68 ± 0.00 ^g^	5.71 ± 0.00 ^h^	6.98 ± 0.00 ^n^	5.56 ± 0.00 ^e^	6.04 ± 0.00 ^i^	4.81 ± 0.00 ^a^	5.64 ± 0.00 ^f^	5.31 ± 0.00 ^d^
OOO	21.30 ± 0.02 ^b^	26.14 ± 0.00 ^m^	22.90 ± 0.02 ^e^	23.28 ± 0.00 ^g^	19.18 ± 0.01 ^a^	23.97 ± 0.02 ^h^	23.10 ± 0.00 ^f^	22.86 ± 0.02 ^e^	21.62 ± 0.01 ^c^	24.12 ± 0.01 ^i^	25.22 ± 0.00 ^k^	24.61 ± 0.01 ^j^	25.75 ± 0.00 ^l^	24.16 ± 0.02 ^i^	21.65 ± 0.00 ^d^
SLO	3.24 ± 0.00 ^a^	4.27 ± 0.00 ^l^	4.14 ± 0.00 ^k^	3.65 ± 0.00 ^e^	3.93 ± 0.00 ^i^	3.40 ± 0.00 ^b^	3.68 ± 0.00 ^f^	3.68 ± 0.00 ^f^	4.29 ± 0.00 ^m^	3.55 ± 0.00 ^c^	4.15 ± 0.00 ^k^	4.05 ± 0.00 ^j^	3.62 ± 0.00 ^d^	3.75 ± 0.00 ^g^	3.88 ± 0.00 ^h^
OLO	1.42 ± 0.00 ^g^	1.93 ± 0.00 ^l^	0.89 ± 0.00 ^b^	1.20 ± 0.00 ^e^	0.77 ± 0.00 ^a^	2.10 ± 0.00 ^m^	1.09 ± 0.00 ^c^	1.66 ± 0.00 ^i^	1.51 ± 0.00 ^h^	1.94 ± 0.00 ^l^	1.87 ± 0.00 ^k^	1.22 ± 0.00 ^f^	1.65 ± 0.00 ^i^	1.76 ± 0.00 ^j^	1.13 ± 0.00 ^d^
SOA	2.89 ± 0.00 ^a^	3.67 ± 0.02 ^g^	3.49 ± 0.02 ^c^	3.56 ± 0.01 ^e^	3.54 ± 0.00 ^e^	3.92 ± 0.01 ^j^	3.63 ± 0.00 ^f^	3.44 ± 0.00 ^b^	3.52 ± 0.00 ^c,d^	4.04 ± 0.00 ^k^	3.86 ± 0.02 ^i^	3.85 ± 0.00 ^i^	3.71 ± 0.01 ^h^	4.12 ± 0.01 ^l^	3.45 ± 0.00 ^b^
AOO	2.29 ± 0.00 ^a^	3.67 ± 0.03 ^l^	2.78 ± 0.02 ^f^	2.39 ± 0.00 ^b^	2.30 ± 0.00 ^a^	3.15 ± 0.01 ^j^	2.66 ± 0.00 ^d^	3.09 ± 0.00 ^i^	2.59 ± 0.00 ^c^	3.00 ± 0.00 ^h^	4.05 ± 0.02 ^m^	2.97 ± 0.00 ^g^	4.08 ± 0.01 ^m^	3.54 ± 0.01 ^k^	2.75 ± 0.00 ^e^

^1^ Values are means ± SD. Different letters in a column indicate significant differences (*p* < 0.05). * O: Oleic acid; P: Palmitic acid; L: Linoleic acid; S: Stearic acid; P_O_: Palmitoleic acid; A: Arachidic acid; M: Myristic acid.

**Table 4:** The minor components content (mg/kg of oil) and antioxidant capacity (μmol Vitamin E /kg) of oils extracted from 15 macadamia cultivars ^1^.

No.	Antioxidant Capacity			Polyphenols	α-Tocotrienols	Squalene	Minerals			
	DPPH *	ABTS	FRAP				Mg	Ca	K	Na
1	126.64 ± 9.76 ^a^	255.17 ± 5.40 ^a^	136.26 ± 0.92 ^a^	19.74 ± 1.83 ^a^	52.3 ± 1.11 ^k^	91.15 ± 1.38 ^a^	12.21 ± 0.51 ^c^	N.D. ^a^	22.28 ± 0.20 ^d^	1.20 ± 0.01 ^h^
2	359.36 ± 5.67 ^g^	1086.94 ± 18.05 ^i,j^	324.72 ± 3.42 ^k^	123.40 ± 1.67 ^k^	46.6 ± 0.26 ^i^	268.08 ± 1.52 ^n^	17.05 ± 0.08 ^f^	N.D. ^a^	19.42 ± 0.16 ^d^	0.75 ± 0.02 ^d^
3	303.80 ± 7.51 ^f^	1036.79 ± 33.73 ^i^	246.63 ± 0.69 ^h^	77.17 ± 2.67 ^h^	33.5 ± 0.25 ^d^	252.07 ± 0.59 ^l^	15.54 ± 0.65 ^e^	1.35 ± 0.01 ^c^	17.14 ± 0.46 ^b^	0.86 ± 0.01 ^e^
4	247.50 ± 9.76 ^d,e^	629.39 ± 21.88 ^f^	207.76 ± 2.28 ^f^	52.64 ± 1.33 ^e^	46.9 ± 0.26 ^i^	232.13 ± 0.66 ^i^	8.84 ± 0.09 ^b^	N.D. ^a^	18.58 ± 0.33 ^c^	0.82 ± 0.02 ^e^
5	307.56 ± 1.30 ^f^	1083.09 ± 28.29 ^ij^	270.58 ± 1.39 ^j^	96.16 ± 3.50 ^j^	30.0 ± 0.72 ^b^	264.07 ± 0.79 ^m^	14.00 ± 0.18 ^d^	1.21 ± 0.00 ^b^	24.81 ± 1.46 ^e^	0.61 ± 0.02 ^c^
6	179.19 ± 19.42 ^b,c^	438.81 ± 9.85 ^d^	160.56 ± 1.59 ^c^	50.17 ± 1.50 ^d,e^	33.7 ± 0.17 ^d^	163.89 ± 0.64 ^d^	14.42 ± 0.19 ^d^	1.32 ± 0.01 ^c^	17.88 ± 0.45 ^b^	1.05 ± 0.06 ^f^
7	171.68 ± 14.02 ^b^	339.28 ± 19.01 ^c^	158.13 ± 3.31 ^c^	40.97 ± 0.50 ^c^	35.6 ± 0.00 ^e^	182.79 ± 0.95 ^e^	8.13 ± 0.11 ^a^	N.D. ^a^	13.75 ± 0.44 ^a^	0.55 ± 0.02 ^c^
8	155.91 ± 8.36 ^b^	326.16 ± 2.31 ^c^	156.74 ± 2.11 ^c^	24.22 ± 1.83 ^b^	27.9 ± 0.29 ^a^	158.62 ± 0.64 ^c^	18.94 ± 0.26 ^g^	1.44 ± 0.02 ^d^	23.77 ± 0.83 ^e^	0.55 ± 0.01 ^c^
9	191.20 ± 26.72 ^b,c^	520.60 ± 19.69 ^e^	177.56 ± 0.35 ^d^	40.97 ± 1.83 ^c^	53.1 ± 1.21 ^k^	189.79 ± 0.68 ^f^	22.25 ± 0.45 ^h^	1.55 ± 0.01 ^e^	23.41 ± 0.40 ^e^	0.41 ± 0.02 ^a^
10	225.73 ± 16.92 ^d^	618.59 ± 13.18 ^f^	183.11 ± 2.11 ^e^	50.40 ± 0.17 ^d^	45.1 ± 0.87 ^h^	229.22 ± 0.74 ^h^	15.65 ± 0.47 ^e^	N.D. ^a^	21.38 ± 0.88 ^d^	0.51 ± 0.01 ^b^
11	221.23 ± 13.28 ^d^	579.24 ± 34.13 ^a^	178.26 ± 1.59 ^d^	50.28 ± 0.67 ^de^	32.2 ± 0.52 ^c^	208.77 ± 0.39 ^g^	49.90 ± 0.70 ^l^	28.50 ± 0.88 ^i^	48.30 ± 2.33 ^i^	1.01 ± 0.03 ^f^
12	259.51 ± 6.00 ^e^	669.52 ± 1.54 ^g^	227.89 ± 4.59 ^g^	55.59 ± 1.17 ^f^	40.9 ± 0.21 ^g^	240.16 ± 0.69 ^j^	46.10 ± 0.14 ^k^	21.00 ± 0.56 ^h^	51.80 ± 0.90 ^i^	2.51 ± 0.01 ^k^
13	300.80 ± 16.90 ^f^	819.20 ± 14.88 ^h^	234.48 ± 6.10 ^g^	64.43 ± 0.67 ^g^	38.3 ± 0.51 ^f^	244.71 ± 0.66 ^k^	34.40 ± 0.16 ^i^	17.70 ± 0.22 ^f^	34.90 ± 0.17 ^g^	1.85 ± 0.00 ^j^
14	153.66 ± 22.91 ^a,b^	280.64 ± 26.77 ^a,b^	143.55 ± 3.08 ^b^	19.74 ± 1.83 ^a^	52.3 ± 1.11 ^k^	91.15 ± 1.38 ^a^	38.10 ± 0.29 ^j^	18.70 ± 0.14 ^g^	43.90 ± 0.17 ^h^	1.17 ± 0.00 ^g^
15	306.81 ± 10.90 ^f^	1039.11 ± 8.13 ^i^	263.64 ± 3.66 ^i^	123.40 ± 1.67 ^k^	46.6 ± 0.26 ^i^	268.08 ± 1.52 ^n^	33.40 ± 1.14 ^i^	18.20 ± 0.21 ^g^	32.30 ± 1.54 ^f^	1.59 ± 0.02 ^i^
Max	359.36 ± 5.67 ^g^	1086.94 ± 18.05 ^i,j^	324.72 ± 3.42 ^k^	123.40 ± 1.67 ^k^	53.1 ± 1.21 ^k^	268.08 ± 1.52 ^n^	49.90 ± 0.70 ^l^	28.50 ± 0.88 ^i^	51.80 ± 0.90 ^i^	2.51 ± 0.01 ^k^
Min	126.64 ± 9.76 ^a^	255.17 ± 5.40 ^a^	136.26 ± 0.92 ^a^	19.74 ± 1.83 ^a^	27.9 ± 0.29 ^a^	91.15 ± 1.38 ^a^	8.13 ± 0.11 ^a^	N.D. ^a^	13.75 ± 0.44 ^a^	0.41 ± 0.02 ^a^

^1^ Values are means ± SD. Different letters in a column indicate significant differences (*p* < 0.05). N.D., not detected. * DPPH: 2,2-diphenyl-1-picrylhydrazyl radical scavenging ability. ABTS: 2,2′-azino-bis (3-ethylbenzothiazoline-6-sulfonic acid) diammonium salt radical scavenging ability. FRAP: ferric reducing ability of plasma.

**Table 5:** Phytosterols content (mg/kg of oil) of oils extracted from 15 macadamia cultivars ^1^.

No.	Campesterol	Clerosterol	β-Sitosterol	β-Stiostanol	Δ^5^- Avenasterol	Δ^5,24^- Stigmastadienol	Δ^7^- Stigmastenol	Δ^7^- Avenasterol	Total Phytosterols
1	88.36 ± 1.13 ^c^	15.50 ± 0.77 ^c,d^	1533.93 ± 20.45 ^f^	8.66 ± 0.49 ^e^	291.75 ± 2.80 ^j^	12.27 ± 0.74 ^d^	30.71 ± 0.25 ^f^	25.59 ± 0.25 ^g^	2006.77 ± 19.65 ^d^
2	103.45 ± 1.85 ^g^	12.88 ± 0.60 ^a,b^	1248.78 ± 23.80 ^a^	9.59 ± 0.83 ^e^	170.01 ± 0.65 ^a^	9.39 ± 0.54 ^b^	7.82 ± 0.07 ^a^	7.58 ± 0.10 ^a^	1569.50 ± 25.50 ^a^
3	88.58 ± 0.62 ^c^	11.69 ± 0.50 ^a^	1354.52 ± 15.65 ^b^	7.29 ± 0.09 ^c,d^	263.07 ± 0.84 ^g^	7.96 ± 0.21 ^a^	18.09 ± 0.38 ^c,d^	16.19 ± 0.16 ^c^	1767.39 ± 15.69 ^a,b^
4	82.36 ± 0.84 ^a,b^	16.26 ± 0.13 ^d^	1613.7 ± 32.24 ^g^	6.94 ± 0.15 ^c^	250.74 ± 1.40 ^e^	10.96 ± 0.38 ^c,d^	18.60 ± 0.27 ^d^	15.30 ± 0.27 ^b^	2014.86 ± 34.65 ^d^
5	81.45 ± 0.78 ^a^	11.41 ± 0.31 ^a^	1357.07 ± 35.69 ^b^	6.22 ± 0.02 ^b^	343.78 ± 2.11 ^l^	10.17 ± 0.70 ^b,c^	34.95 ± 0.05 ^g^	25.64 ± 0.54 ^g^	1870.69 ± 36.51 ^b,c^
6	114.13 ± 1.39 ^i^	14.39 ± 0.52 ^b,c^	1422.23 ± 17.78 ^c,d^	8.84 ± 0.65 ^e^	338.72 ± 1.20 ^k^	14.61 ± 0.21 ^e^	35.93 ± 0.14 ^h^	32.11 ± 0.24 ^h^	2014.28 ± 15.60 ^d^
7	93.57 ± 0.05 ^d^	13.85 ± 0.23 ^b^	1470.71 ± 17.61 ^d,e^	7.13 ± 0.32 ^c^	191.22 ± 0.41 ^c^	10.63 ± 0.76 ^c,d^	30.73 ± 0.25 ^f^	19.54 ± 0.05 ^e^	1837.38 ± 17.64 ^b^
8	97.75 ± 0.78 ^f^	14.33 ± 0.34 ^b,c^	1441.08 ± 10.47 ^d^	7.74 ± 0.28 ^c^	266.99 ± 3.81 ^g,h^	12.35 ± 0.55 ^d^	17.96 ± 0.06 ^c^	19.13 ± 0.09 ^d^	1877.33 ± 14.87 ^b,c^
9	95.86 ± 0.15 ^e^	13.48 ± 0.66 ^b^	1443.81 ± 21.86 ^d^	6.97 ± 0.27 ^c^	362.08 ± 1.79 ^g^	19.79 ± 0.80 ^h^	47.95 ± 0.19 ^j^	41.34 ± 0.49 ^i^	2031.28 ± 22.79 ^d^
10	90.98 ± 1.42 ^c^	13.49 ± 0.60 ^b^	1510.38 ± 14.65 ^f^	8.34 ± 0.11 ^e^	333.57 ± 2.80 ^d^	18.38 ± 0.36 ^g^	48.57 ± 0.61 ^j^	40.83 ± 0.08 ^i^	2064.54 ± 15.06 ^d^
11	107.41 ± 0.95 ^h^	13.39 ± 0.24 ^b^	1441.48 ± 16.65 ^d^	7.74 ± 0.12 ^d^	257.25 ± 2.37 ^f^	15.92 ± 0.35 ^f^	47.42 ± 0.29 ^j^	31.40 ± 0.35 ^h^	1922.02 ± 16.98 ^c,d^
12	93.72 ± 0.21 ^d^	11.71 ± 0.42 ^a^	1329.66 ± 15.40 ^b^	7.62 ± 0.20 ^d^	178.90 ± 0.65 ^b^	9.37 ± 0.48 ^b^	30.49 ± 0.73 ^f^	20.69 ± 0.87 ^f^	1682.16 ± 15.02 ^a^
13	91.40 ± 1.96 ^c,d^	12.63 ± 0.53 ^ab^	1396.40 ± 6.51 ^c^	7.72 ± 0.05 ^d^	280.42 ± 2.73 ^i^	9.02 ± 0.19 ^b^	16.55 ± 0.12 ^b^	16.72 ± 0.43 ^c^	1830.86 ± 6.34 ^b^
14	92.06 ± 0.44 ^d^	13.10 ± 0.61 ^b^	1324.06 ± 9.15 ^b^	7.22 ± 0.08 ^c^	248.04 ± 1.01 ^e^	7.84 ± 0.32 ^a^	24.45 ± 0.40 ^e^	21.12 ± 0.65 ^f^	1737.89 ± 11.44 ^a,b^
15	83.90 ± 0.64 ^b^	12.41 ± 0.40 ^a^	1348.70 ± 28.15 ^b^	5.39 ± 0.27 ^a^	272.42 ± 1.17 ^h^	12.82 ± 1.70 ^d^	39.17 ± 0.26 ^i^	31.83 ± 0.11 ^h^	1806.64 ± 32.35 ^b^
Max	114.13 ± 1.39 ^i^	16.26 ± 0.13 ^d^	1613.7 ± 32.24 ^g^	9.59 ± 0.83 ^e^	362.08 ± 1.79 ^g^	18.38 ± 0.36 ^g^	48.57 ± 0.61 ^j^	41.34 ± 0.49 ^i^	2064.54 ± 15.06 ^d^
Min	81.45 ± 0.78 ^a^	11.41 ± 0.31 ^a^	1248.78 ± 23.80 ^a^	5.39 ± 0.27 ^a^	170.01 ± 0.65 ^a^	7.84 ± 0.32 ^a^	7.82 ± 0.07 ^a^	7.58 ± 0.10 ^a^	1569.50 ± 25.50 ^a^

^1^ Values are means ± SD. Different letters in a column indicate significant differences (*p* < 0.05).

**Table 6:** Correlations between minor components and antioxidant capacity.

	Polyphenols	Tocotrienols	Squalence	Campesterol	Clerosterol	β-Sitosterol	β-Stiostanol	Δ^5^- Avenasterol	Δ^5,24^- Stigmastadienol	Δ^7^- Stigmastenol	Δ^7^- Avenasterol
DPPH	0.939 ^a^	−0.111	0.927 ^a^	−0.225	−0.605 ^b^	−0.525 ^b^	−0.144	−0.219	−0.327	−0.307	−0.348
FRAP	0.965 ^a^	−0.060	0.870 ^a^	−0.217	−0.584 ^b^	−0.589 ^b^	−0.106	−0.266	−0.354	−0.360	−0.397
ABTS	0.938 ^a^	−0.142	0.899 ^a^	−0.287	−0.647 ^a^	−0.519 ^b^	−0.242	−0.063	−0.260	−0.198	−0.226

^a^ Correlation is significant at the 0.01 level (2-tailed). ^b^ Correlation is significant at the 0.05 level (2-tailed).

**Table 7:** Equation, variable, and regression coefficient in the prediction of the antioxidant capacity by MLR.

Dependent Variable	*R* ^2^	Adjusted *R*^2^	Variable	*R*	Standard Error	t	Significance (Two Tails *p*) ^a^	Equation
DPPH	0.946	0.937	(Constant) polyphenols squalene	35.671	20.695	1.724	0.110	Y = 35.671 + 0.546 (polyphenols) + 0.467 (squalene)
				0.546	0.306	4.374	0.001	
				0.467	0.158	3.741	0.003	
ABTS	0.920	0.907	(Constant) polyphenols squalene	−142.676	106.934	−1.334	0.207	Y = −142.676 + 0.621 (polyphenols) + 0.376 (squalene)
				0.621	1.583	4.111	0.001	
				0.376	0.816	2.491	0.028	
FRAP	0.932	0.927	(Constant) polyphenols	99.412	8.769	11.337	0.000	Y = 99.412 + 0.965 (polyphenols)
				0.965	0.138	13.346	0.000	

^a^ *p* < 0.05, significant regression.

## Data Availability

Data is contained within the article.
